# Theoretical Investigation of the Prospect to Tailor ZnO Electronic Properties with VP Thin Films

**DOI:** 10.3390/nano11061412

**Published:** 2021-05-27

**Authors:** Anastasiia S. Kholtobina, Evgenia A. Kovaleva, Julia Melchakova, Sergey G. Ovchinnikov, Alexander A. Kuzubov

**Affiliations:** 1 Materials Science and Engineering, Industrial Engineering & Management School, KTH Royal Institute of Technology, Brinellvågen 23, 11428 Stockholm, Sweden; akholtobina93@mail.ru; 2 Kirensky Institute of Physics, Federal Research Center KSC Siberian Branch, Russian Academy of Sciences, Akademgorodok 50, bld. 38, 660036 Krasnoyarsk, Russia; sgo@iph.krasn.ru (S.G.O.); alexxkuzubov@gmail.com (A.A.K.); 3 Faculty of Physics, Tomsk State University, 36 Lenin Ave, 634050 Tomsk, Russia; iuliia.melchakova@gmail.com

**Keywords:** ZnO, vanadium phosphide, thin films, nanocomposite, photocatalysts, density functional theory

## Abstract

The atomic and electronic structure of vanadium phosphide one- to four-atomic-layer thin films and their composites with zinc oxide substrate are modelled by means of quantum chemistry. Favorable vanadium phosphide to ZnO orientation is defined and found to remain the same for all the structures under consideration. The electronic structure of the composites is analyzed in detail. The features of the charge and spin density distribution are discussed.

## 1. Introduction

Zinc oxide has been of particular interest to the researchers during the last several decades due to its chemical stability and non-toxicity along with the low cost. This material is promising for a number of potential applications such as photoelectric elements [1,2,3,4,5,6,7], light-emitting diodes (LEDs) [8,9,10,11,12], gas sensors [13,14,15], biosensors [16], photodetectors [17,18] and photocatalytic devices [19,20].

Electronic properties of ZnO are strongly affected by the synthesis conditions and method. This fact is associated with the point defects (oxygen/zinc vacancy and oxygen/zinc interstitials) acting as dopants and influencing physical and chemical characteristics of material [21,22,23,24]. ZnO doping enhances its physical properties—namely, electric conductivity [25], and transparency [26]—and decreases the electron work function [27]. Ferromagnetic properties [28,29] may also occur in doped ZnO while the pristine material is non-magnetic.

n-doping of ZnO is usually reached by XIII group elements (i.e., B [30], Al [31,32], Ga [33,34], In [35]) as well as transition metals such as Ti [36]. On the other hand, XV group elements (N [37,38], P [39,40] and Sb [41]) are promising p-type dopants substituting oxygen atoms in ZnO structure. ZnO doped by transition metal atoms arouses great interest due to the opportunity to obtain diluted magnetic semiconductors (DMS) for new device applications.

Besides the doping of ZnO with different elements of periodic table, the formation of thin films-based composites is another popular way to tune its properties. For instance, synthesis and enhanced photocatalytic properties have been recently reported for ZnO-based composites with graphene [42,43,44,45]. Another way to improve ZnO photocatalytic activity is using MXenes, a promising family of materials defined by M_n+1_X_n_T_x_ composition where M is an early transition metal, X is carbon and/or nitrogen atom and T represents the surface-terminating functional groups [46,47,48]. Thus, investigations of zinc oxide-based metamaterials obtained by its doping as well as growing thin films of transition metals compounds on ZnO substrate are a promising direction of modern materials science.

Transition metal phosphides (TMP), one more promising family of two-dimensional transition metal compounds, have gained significant research interest due to their unique properties and catalytic activity in hydrogen evolution reaction [49,50,51,52,53]. Some of them have even been predicted to be comparable with Pt (111) surface [54]. Extensive theoretical studies of M_2_P monolayers have shown them as promising candidates for catalysis and electrode materials [55,56,57]. The most recent study of tetragonal VP monolayer reveals its half-metallicity and interesting optical properties [58].

The present paper aims to show how ZnO electronic structure changes when forming nanoscale composites with VP thin films. First, thin films of vanadium phosphide with various thickness and composition are characterized by means of density functional theory. After that, ZnO/VP stacking, electronic and magnetic properties are discussed.

## 2. Computational Methods

All quantum chemical calculations were performed within the framework of density functional theory using the plane wave basis set and projector-augmented wave method [59,60], as implemented in Vienna Ab-initio Simulation Package [61,62,63,64]. GGA-PBE spin-polarized exchange-correlation functional [65] and Grimme correction [66] for van der Waals interactions were used for electronic and structural optimization. The residual forces acting on atoms being less than 10^−3^ eV/Å were used as stopping criteria for cell vectors and geometry optimization. Monkhorst-Pack *k*-point first Brilloin zone sampling [67] was used with *k*-point mesh containing 12 × 12 × 6 points along three translation vectors for bulk ZnO and VP calculations. When calculating the slabs and interfaces, the vacuum interval of 15 Å was used to guarantee the absence of interactions between slab images in periodic boundary conditions. For these structures, 12 × 12 × 1 *k*-point mesh was used.

The surface energy for all slabs was estimated as:

(1) $$E_{surf}=(E_{sc}-n\cdot E_{uc})/(2\cdot S)$$

where $E_{surf}$, $E_{sc}$, $E_{uc}$, *n*, *S* correspond to the surface energy, total energy of the surface supercell, total energy of ZnO unit cell, number of unit cells in the supercell, and the area of ZnO slab unit cell, respectively.

The most favorable orientation of VP slab with respect to ZnO surface was determined by comparing stacking energies of each configuration estimated using the equation:

*E_stack_* = *E_comp_* − *E_ZnO_* − *E_VP_* (2)

where *E_comp_*, *E_ZnO_*, *E_VP_* correspond to the total energies of composite, pure ZnO slab and pure VP slab, respectively.

## 3. Results and Discussions

At the first step, the correspondence of the ZnO (0001) surface and vanadium phosphide hexagonal lattices was proved. The zinc oxide hexagonal unit cell belongs to the space group P6_3_mc with lattice parameters *a* = *b* = 3.25, *c =* 5.21 Å [68] while the VP hexagonal unit cell belongs to the space group P6_3_/mmc with lattice parameters *a = b =* 3.180, *c =* 6.220 Å [69]. A set of free-standing ZnO (0001) slabs with the number of atomic layers varying from 7 to 12 were modelled. It was found that the values of *E_surf_* are close to each other and lie in the range of 1.854 to 1.883 J/m^2^. Thus, the one with the smallest number of atoms was used as the surface unit cell for further calculations. Next, VP slabs cut from the bulk crystal with the number of layers decreasing from four to one were modelled.

**Figure 1:**
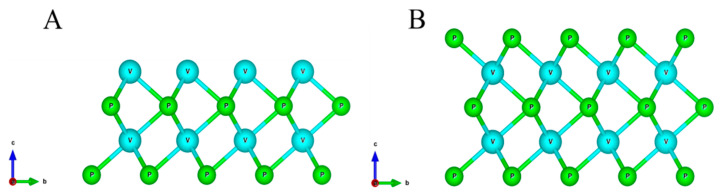
Atomic structure of VP thin films. (**A**) stoichiometric (V_2_P_2_) and (**B**) non-stoichiometric (V_2_P_3_) VP bilayer.

Lattice parameter *a* as well as the corresponding magnetic moments for VP are presented in Table 1. Structural parameters of bulk VP are in good agreement with experimental data [69]. The structure stoichiometries correspond to the number of each element’s atomic layers. Thin films of two or more layers are close to the original bulk structure while monolayers demonstrate fluctuations of a parameter which can be explained in terms of structural instability. The stoichiometric compositions of VP thin films are characterised by larger magnetic moments on vanadium atoms caused by the V dangling bonds while their non-stoichiometric counterparts have magnetic moments close to zero (Figure 1 illustrates atomic structure for stoichiometric and non-stoichiometric bilayer of VP). In this work, we mainly focus on stoichiometric structures as V-terminated surfaces possessing larger magnetic moments. Magnetic catalysts are considered to be environmentally friendly as they can be easily and completely separated from reactants using an external magnet without any loss, unlike other heterogeneous catalysts requiring filtration, centrifugation and other techniques that might be quite sophisticated [70]. It is also known that not only charge transfer but also spin transfer may occur when the molecule is adsorbed on magnetic surface, enhancing its catalytic properties [71] and expanding the area of potential applications in spintronic devices [72]. Non-stoichiometric ones are presented both for the reference and as an intermediate step of thin films formation.

**Figure 2:**
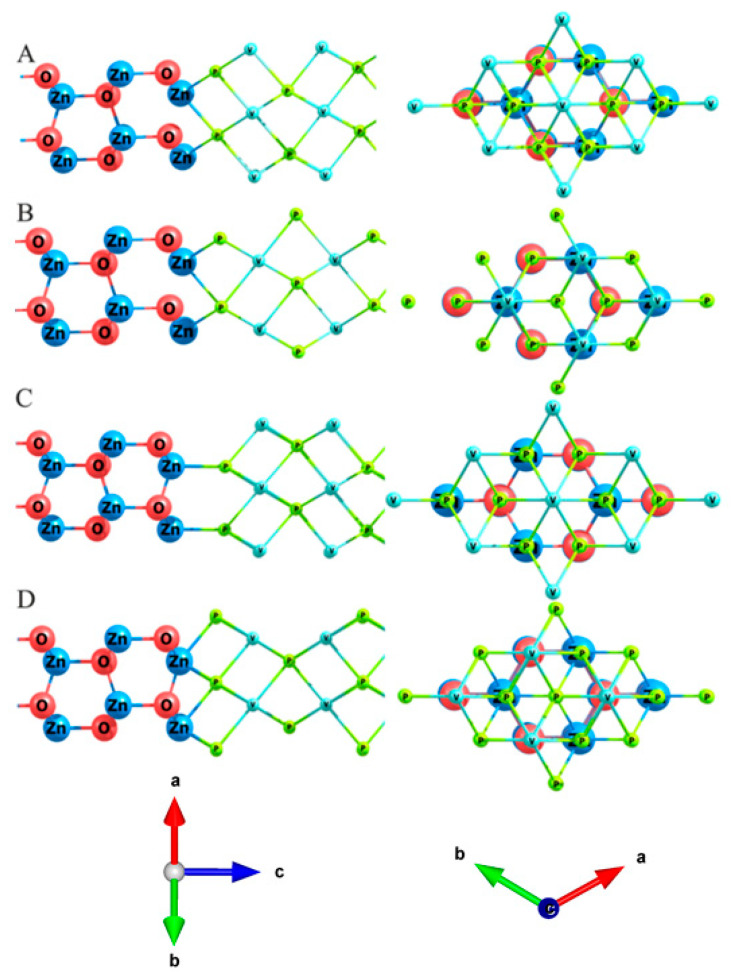
VP slab orientation with respect to ZnO in VP/ZnO composites. (**A**) [P_top_O:V_hex]; (**B**) [P_top_O:P_hex]; (**C**) [P_top_Zn:V_hex]; (**D**) [V_top_O:P_hex].

The manifold of composite structures considered included different VP film orientations with respect to ZnO (see Figure 2 for the notations: A_top_B corresponds to the atom A of VP being on top of the atom B of ZnO; A_hex represents hexagonal hollow site below the atom A of VP).

The [P_top_Zn:V_hex] configuration of VP/ZnO composite was found to have the lowest stacking energy for both V_4_P_4_/ZnO and VP monolayer/ZnO structures (−1.273 eV and −1.167 eV, respectively, see Table 2). This configuration is also characterized by the largest values of magnetic moments, and the VP monolayer possesses the largest among all (2.285 µ_B_). According to the common trend in stacking energies for one- and four-layer VP films, only [P_top_Zn:V_hex] configuration was constructed for two- and three-layer ones.

**Figure 3:**
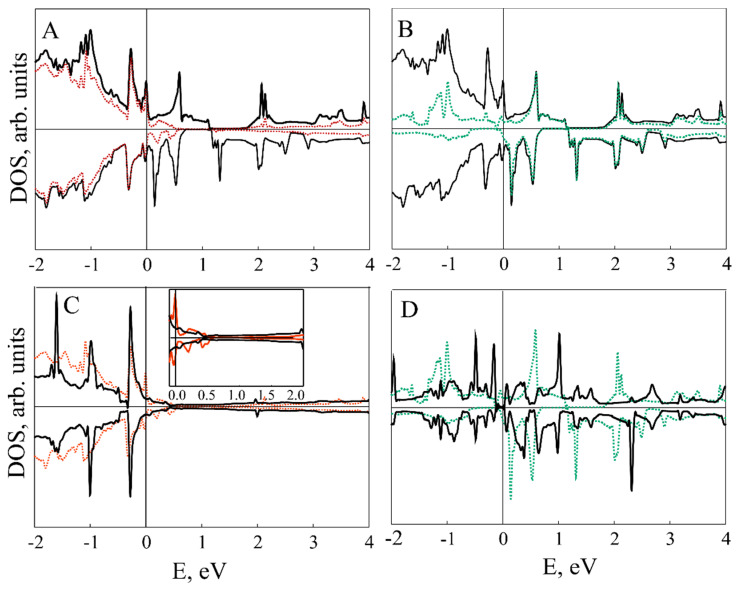
DOS for VP monolayer/ZnO composite. (**A**) Black and red lines correspond to composite TDOS and ZnO PDOS; (**B**) black and green lines correspond to composite TDOS and VP monolayer PDOS; (**C**) black and red lines correspond to TDOS of pristine ZnO slab and PDOS of ZnO fragment in VP monolayer/ZnO composite; (**D**) black and green lines correspond to TDOS of pristine VP monolayer and PDOS of VP in the composite structure, respectively.

**Figure 4:**
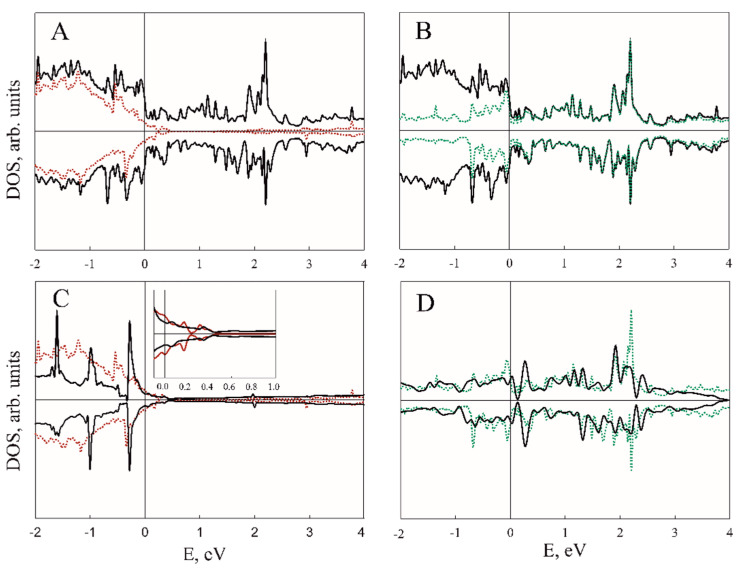
DOS for V_3_P_3_/ZnO composite. (**A**) Black and red lines correspond to composite TDOS and ZnO PDOS; (**B**) black and green lines correspond to composite TDOS and VP PDOS; (**C**) black and red lines correspond to TDOS of pristine ZnO slab and PDOS of ZnO fragment in V_3_P_3_/ZnO composite; (**D**) black and green lines correspond to TDOS of pristine V_3_P_3_ slab and PDOS of VP in the composite structure, respectively.

**Figure 5:**
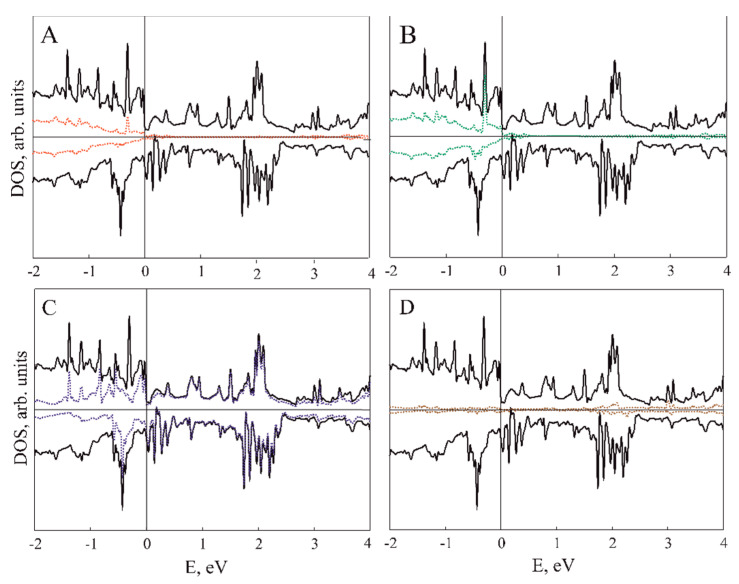
DOS for V_4_P_4_/ZnO composite. Black, red (**A**), green (**B**), blue (**C**) and brown (**D**) lines corresponds to composite TDOS and Zn, O, V and P atoms PDOS, respectively.

**Figure 6:**
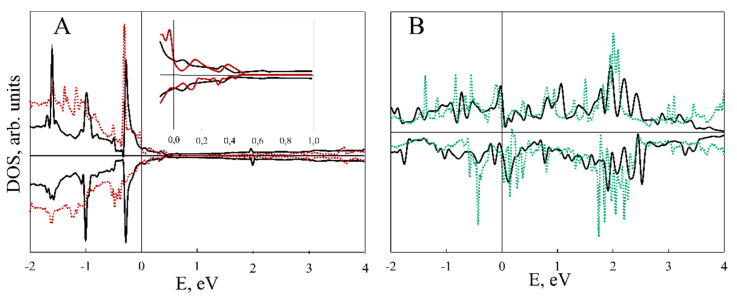
DOS for V_4_P_4_/ZnO composite. (**A**) Black and red lines correspond to TDOS of pristine ZnO slab and PDOS of ZnO fragment in V_4_P_4_/ZnO composite; (**B**) black and green lines correspond to TDOS of pristine V_4_P_4_ slab and PDOS of VP in the composite structure, respectively.

Figure 3, Figure 4, Figure 5 and Figure 6 illustrate the total (TDOS) and partial (PDOS) densities of states for the VP/ZnO composites in favorable configuration. As can be clearly seen from A and B parts of Figure 3 and Figure 4, the ZnO slab mostly contributes to the states in the valence zone while the conduction zone is formed predominately by VP film. Analysis of C and D parts of the same figures shows how the slabs affect each other in comparison with isolated ZnO and VP thin films.

Composite formation leads to the shifting and broadening of DOS peaks, which is more prominent for the VP monolayer in VP/ZnO composite while VP thickness up to three layers leads to the change mostly in the ZnO valence zone (see Figure 4). However, the levels of zinc oxide thin film above the Fermi level are much less affected (see insets in Figure 3C and Figure 4C).

Figure 5 demonstrates element-resolved PDOS for V_4_P_4_/ZnO structure. While Zn and O states are highly hybridized, V contribution is dominating for VP and PDOS of P are almost negligible. Figure 6, similarly to Figure 3C,D, demonstrates more prominent redistribution of ZnO valence band states and less that of its conduction band.

For the reference, non-stoichiometric configurations of one and three-layer thick VP/ZnO hybrid structure were modelled (see Table 3). The calculated stacking energies revealed that favorable configuration of VP and ZnO slabs’ mutual arrangement remains the same ([P_top_Zn:V_hex]). These values, however, should not be compared to those obtained for stoichiometric structures directly as uniform adsorption of a whole P layer is required to turn from one to another.

**Figure 7:**
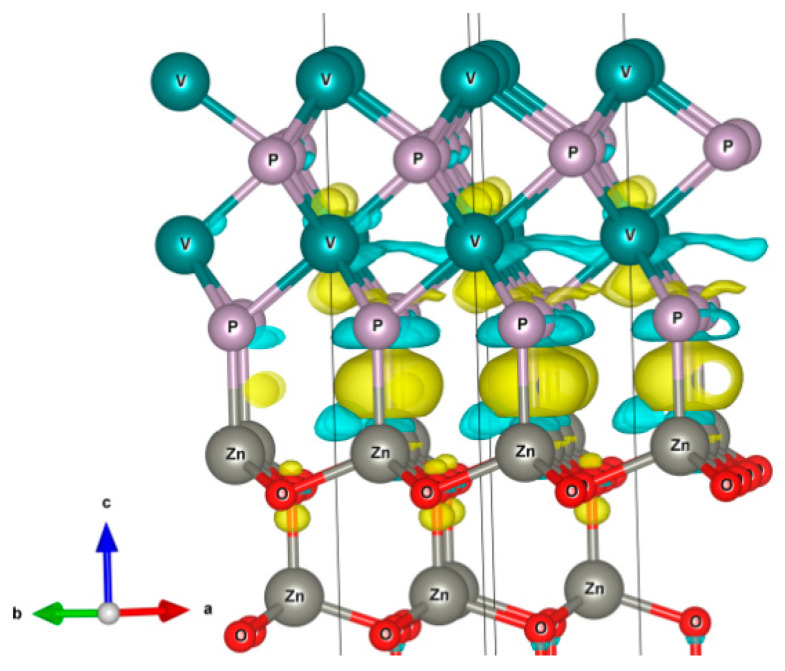
Charge density distribution in V_2_P_2_/ZnO composite. Blue and yellow areas correspond to the lack and excess of charge, respectively.

In addition, the charge and spin density distributions were analyzed. The negative charge on VP slab demonstrates the electron transferred to it from the ZnO slab (Figure 7).

The amount of charge transfer estimated by the AIM (Bader) method [73,74,75] is listed in Table 4. The same non-uniform trend is observed for both charge and spin distribution as the number of layers increases. The latter is generally in agreement with values calculated for pristine VP slabs.

**Figure 8:**
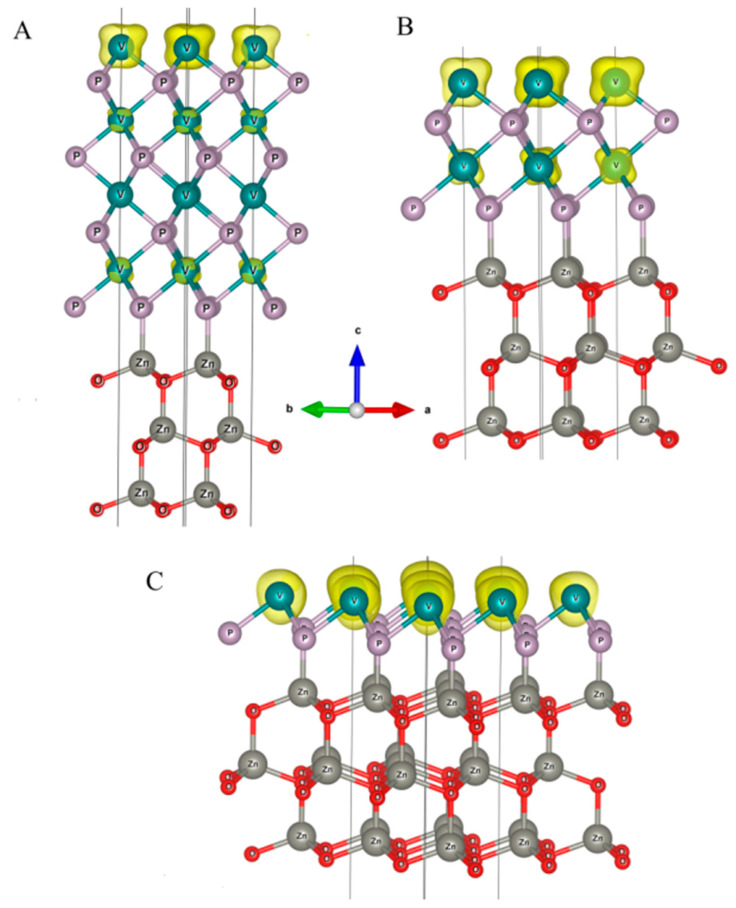
Spin density distribution in VP/ ZnO composites with four (**A**), two (**B**) and one layer (**C**) of VP.

According to Figure 8, which demonstrates spin density spatial distribution, the topmost V layer gains the most of the magnetic moment while the magnetism in deeper-lying V atoms is rather quenched with the increase in the number of VP layers in the composite.

## 4. Conclusions

The atomic and electronic structure of VP thin films was calculated and the possibility of VP/ZnO composite formation was proven by quantum chemical modelling. Configuration characterized by phosphorous atoms being atop the Zn ones and vanadium atoms placed above the hexagon centre was found to be favourable for all structures considered regardless of the number of VP layers and stoichiometry of structure. The valence band is mostly formed by the ZnO slab while VP states are more prominent in the conduction band. Zinc and oxygen states are highly hybridized whereas VP DOS rises mainly from vanadium atoms. The topmost V atoms are visibly spin-polarized which opens opportunities for various applications of these structures in spintronics as magnetic substrates for organic molecules or metal complexes adsorption and in catalysis as magnetic catalysts that can be removed from the solution with external magnet. These applications are to be further investigated.

## Figures and Tables

**Table 1:** Lattice parameter a, magnetic moment and stability of stoichiometric and non-stoichiometric configurations of VP from one to four layers.

Configuration	Magnetic Moment, μ_B_	a, Å
Bulk VP	0.000	3.130
VP monolayer	2.000	2.962
VP_2_	0.756	3.214
V_2_P_2_	1.636	3.058
V_2_P_3_	0.000	3.058
V_3_P_3_	1.511	3.078
V_3_P_4_	0.147	3.111
V_4_P_4_	1.791	3.086
V_4_P_5_	0.025	3.115

**Table 2:** Stacking energies and magnetic moments for different VP slab orientation in structures with one and four VP layers.

Structure	V_4_P_4_		VP Monolayer	
	*E_stack_*, eV	µ, µ_B_	*E_stack_*, eV	µ, µ_B_
[P_top_O:V_hex]	−0.637	1.159	−0.465	1.390
[P_top_O:P_hex]	−0.644	1.206	−0.655	2.163
[P_top_Zn:V_hex]	−1.273	1.242	−1.167	2.285
[V_top_O:P_hex]	−0.705	1.250	−0.772	

**Table 3:** Stacking energies (*E_stack_*) for non-stoichiometric VP/ZnO composites, eV.

Composite Structure	VP_2_	V_3_P_4_
[P_top_O:V_hex]	−1.861	−3.975
[P_top_O:P_hex]	−1.651	−3.781
[P_top_Zn:V_hex]	−2.204	−4.388
[V_top_O:P_hex]	−1.662	−3.781

**Table 4:** Charge (Q_VP_) and magnetic moment (µ_VP_) of VP slab in VP/ZnO composites according to Bader analysis.

Number of Layers	Q_VP_, e^−^	µ_VP_, µ_B_
4	−0.117	1.896
3	−0.186	1.355
2	−0.177	1.842
1	−0.079	2.254

## References

[1] Galdámez-Martinez A., Santana G., Güell F., Martínez-Alanis P.R., Dutt A. (2020). Photoluminescence of ZnO Nanowires: A Review. Nanomaterials.

[2] Boujnah M., Boumdyan M., Naji S., Benyoussef A., El Kenz A., Loulidi M. (2016). High efficiency of transmittance and electrical conductivity of V doped ZnO used in solar cells applications. J. Alloys Compd..

[3] Zhang Q., Hou S., Li C. (2020). Titanium Dioxide-Coated Zinc Oxide Nanorods as an Efficient Photoelectrode in Dye-Sensitized Solar Cells. Nanomaterials.

[4] Ahmed F., Arshi N., Dwivedi S., Koo B.H., Azam A., Alsharaeh E. (2016). Low temperature growth of ZnO nanotubes for fluorescence quenching detection of DNA. J. Mater. Sci. Mater. Med..

[5] Saleem M., Farooq W.A., Khan M.I., Akhtar M.N., Rehman S.U., Ahmad N., Khalid M., Atif M., AlMutairi M.A., Irfan M. (2019). Effect of ZnO Nanoparticles Coating Layers on Top of ZnO Nanowires for Morphological, Optical, and Photovoltaic Properties of Dye-Sensitized Solar Cells. Micromachines.

[6] Li X., Chen X., Yi Z., Zhou Z., Tang Y., Yi Y. (2019). Fabriction of ZnO Nanorods with Strong UV Absorption and Different Hydrophobicity on Foamed Nickel under Different Hydrothermal Conditions. Micromachines.

[7] Lamson T.L., Khan S., Wang Z., Zhang Y.K., Yu Y., Chen Z.S., Xu H. (2018). Patterned Synthesis of ZnO Nanorod Arrays for Nanoplasmonic Waveguide Applications. Opt. Commun..

[8] Lupan O., Pauporté T., Viana B. (2010). Low-voltage UV-electroluminescence from ZnO-nanowire Array/p-GaN light-emitting diodes. Adv. Mater..

[9] Pauporté T., Lupan O., Zhang J., Tugsuz T., Ciofini I., Labat F., Viana B. (2015). Low-Temperature Preparation of Ag-Doped ZnO Nanowire Arrays, DFT Study, and Application to Light-Emitting Diode. ACS Appl. Mater. Interfaces.

[10] Lupan O., Pauporté T., Le Bahers T., Ciofini I., Viana B. (2011). High Aspect Ratio Ternary Zn_1−x_Cd_x_O Nanowires by Electrodeposition for Light-Emitting Diode Applications. J. Phys. Chem. C.

[11] Li L., Zhang Y., Yan L., Jiang J., Han X., Deng G., Chi C., Song J. (2016). n-ZnO/p-GaN heterojunction light-emitting diodes featuring a buried polarization-induced tunneling junction. AIP Adv..

[12] Rahman M.A., Scott J.A., Gentle A., Phillips M.R., Ton-That C. (2018). A facile method for bright, colour-tunable light-emitting diodes based on Ga-doped ZnO nanorods. Nanotechnology.

[13] Davydova M., Laposa A., Smarhak J., Kromka A., Neykova N., Nahlik J., Kroutil J., Drahokoupil J., Voves J. (2018). Gas-sensing behaviour of ZnO/diamond nanostructures. Beilstein J. Nanotechnol..

[14] Do T.A.T., Ho T.G., Bui T.H., Pham Q.N., Giang H.T., Do T.T., Nguyen D.V., Tran D.L. (2018). Surface-plasmon-enhanced ultraviolet emission of Au-decorated ZnO structures for gas sensing and photocatalytic devices. Beilstein J. Nanotechnol..

[15] Nugraha, Saputro A.G., Agusta M.K., Yuliarto B., Dipojono H.K., Rusydi F., Maezono R. (2017). Selectivity of CO and NO adsorption on ZnO (0002) surfaces: A DFT investigation. Appl. Surf. Sci..

[16] Lin C.F., Kao C.H., Lin C.Y., Chen K.L., Lin Y.H. (2020). NH_3_ Plasma-Treated Magnesium Doped Zinc Oxide in Biomedical Sensors with Electrolyte–Insulator–Semiconductor (EIS) Structure for Urea and Glucose Applications. Nanomaterials.

[17] Bai Z.Q., Liu Z.W. (2017). A broadband photodetector based on Rhodamine B-sensitized ZnO nanowires film. Sci Rep..

[18] Chen C., Zhou P., Wang N., Ma Y., San H. (2018). UV-Assisted Photochemical Synthesis of Reduced Graphene Oxide/ZnO Nanowires Composite for Photoresponse Enhancement in UV Photodetectors. Nanomaterials.

[19] Naghizadeh M., Taher M.A., Tamaddon A.M. (2019). Facile synthesis and characterization of magnetic nanocomposite ZnO/CoFe_2_O_4_ hetero-structure for rapid photocatalytic degradation of imidacloprid. Heliyon.

[20] Qi K., Xing X., Zada A., Li M., Wang Q., Liu S., Lin H., Wang G. (2020). Transition metal doped ZnO nanoparticles with enhanced photocatalytic and antibacterial performances: Experimental and DFT studies. Ceram. Int..

[21] Chen R., Wang J., Luo S., Xiang L., Li W., Xie D. (2020). Unraveling photoexcited electron transfer pathway of oxygen vacancy-enriched ZnO/Pd hybrid toward visible light-enhanced methane detection at a relatively low temperature. Appl. Catal. B Environ..

[22] Wang J., Hu C., Xia Y., Zhang B. (2021). Mesoporous ZnO nanosheets with rich surface oxygen vacancies for UV-activated methane gas sensing at room temperature. Sens. Actuators B Chem..

[23] Khai T.V., Thu L.V., Ha L.T.T., Thanh V.M., Lam T.D. (2018). Structural, optical and gas sensing properties of vertically well-aligned ZnO nanowires grown on graphene/Si substrate by thermal evaporation method. Mater. Charact..

[24] Mondal P., Appani S.K., Sutar D.S., Major S.S. (2021). Effect of oxygen partial pressure on the behavior of Ga-doped ZnO/*p*-Si heterojunction diodes fabricated by reactive sputtering. J. Mater. Sci. Mater. Electron..

[25] Pham A.T.T., Hoang D.V., Nguyen T.H., Le O.K.T., Wong D.P., Kuo J.L., Chen K.H., Phan T.B., Tran V.C. (2021). Hydrogen enhancing Ga doping efficiency and electron mobility in high-performance transparent conducting Ga-doped ZnO films. J. Alloys Compd..

[26] Simanjuntak F.M., Prasad O.K., Panda D., Lin C.A., Tsai T.L., Wei K.H., Tseng T.Y. (2016). Impacts of Co doping on ZnO transparent switching memory device characteristics. Appl. Phys. Lett..

[27] Drewelow G., Reed A., Stone C., Roh K., Jiang Z.T., Truc L.N.T., No K., Park H., Lee S. (2019). Work function investigations of Al-doped ZnO for band-alignment in electronic and optoelectronic applications. Appl. Surf. Sci..

[28] Gao Q., Dai Y., Han B., Zhu W., Li X., Li C. (2019). Enhanced gas-sensitivity and ferromagnetism performances by the Ni-doping induced oxygen vacancies in (Mn, Ni) codoped ZnO nanorods. Appl. Surf. Sci..

[29] Ali N., Vijaya A.R., Khan Z.A., Tarafder K., Kumar A., Wadhwa M.K., Singh B., Ghosh S. (2019). Ferromagnetism from non-magnetic ions: Ag-doped ZnO. Sci. Rep..

[30] Hurma T. (2019). Effect of boron doping concentration on structural optical electrical properties of nanostructured ZnO films. J. Mol. Struct..

[31] Sankar ganesh R., Navaneethan M., Mani G.K., Ponnusamy S., Tsuchiya K., Muthamizhchelvan C., Kawasaki S., Hayakawa Y. (2017). Influence of Al doping on the structural, morphological, optical, and gas sensing properties of ZnO nanorods. J. Alloys Compd..

[32] Ajala F., Hamrouni A., Houas A., Lachheb H., Megna B., Palmisano L., Parrino F. (2018). The influence of Al doping on the photocatalytic activity of nanostructured ZnO: The role of adsorbed water. Appl. Surf. Sci..

[33] Zhou Z.F., Ren G.K., Tan X., Liu R., Liu C., Lin Y.H., Nan C.W. (2018). Enhancing the thermoelectric performance of ZnO epitaxial films by Ga doping and thermal tuning. J. Mater. Chem. A.

[34] Jiang M., He G., Chen H., Zhang Z., Zheng L., Shan C., Shen D., Fang X. (2017). Wavelength-Tunable Electroluminescent Light Sources from Individual Ga-Doped ZnO Microwires. Small.

[35] Bharath S.P., Bangera K.V., Shivakumar G.K. (2018). Enhanced gas sensing properties of indium doped ZnO thin films. Superlattices Microstruct..

[36] Chang L.W., Sung Y.C., Yeh J.W., Shih H.C. (2011). Enhanced optoelectronic performance from the Ti-doped ZnO nanowires. J. Appl. Phys..

[37] Oliveira J.A., Nogueira A.E., Gonçalves M.C.P., Paris E.C., Ribeiro C., Poirier G.Y., Giraldi T.R. (2018). Photoactivity of N-doped ZnO nanoparticles in oxidative and reductive reactions. Appl. Surf. Sci..

[38] Kumari V., Mittal A., Jindal J., Yadav S., Kumar N. (2019). S-, N- and C-doped ZnO as semiconductor photocatalysts: A review. Front. Mater. Sci..

[39] Gayen R.N., Paul R. (2018). Phosphorous doping in vertically aligned ZnO nanorods grown by wet-chemical method. Nano-Struct. Nano-Objects.

[40] Murkute P., Sushama S., Ghadi H., Saha S., Chakrabarti S. (2018). Effects of phosphorus implantation time on the optical, structural, and elemental properties of ZnO thin films and its correlation with the 3.31-eV peak. J. Alloys Compd..

[41] Nasser R., Othmen W.B.H., Elhouichet H. (2019). Effect of Sb doping on the electrical and dielectric properties of ZnO nanocrystals. Ceram. Int..

[42] Joshi B.N., Yoon H., Na S.-H., Choi J.-Y., Yoon S.S. (2014). Enhanced photocatalytic performance of graphene-ZnO nanoplatelet composite thin films prepared by electrostatic spray deposition. Ceram. Int..

[43] Lonkar S.P., Pillai V., Abdala A. (2019). Solvent-free synthesis of ZnO-graphene nanocomposite with superior photocatalytic activity. Appl. Surf. Sci..

[44] Xue B., Zou Y. (2018). High photocatalytic activity of ZnO–graphene composite. J. Colloid Interface Sci..

[45] Nguyen V.N., Tran D.T., Nguyen M.T., Le T.T.T., Ha M.N., Nguyen M.V., Pham T.D. (2018). Enhanced photocatalytic degradation of methyl orange using ZnO/graphene oxide nanocomposites. Res. Chem. Intermed..

[46] Guo J., Legum B., Anasori B., Wang K., Lelyukh P., Gogotsi Y., Randall C.A. (2018). Cold Sintered Ceramic Nanocomposites of 2D MXene and Zinc Oxide. Adv. Mater..

[47] Liu X., Chen C. (2020). Mxene enhanced the photocatalytic activity of ZnO nanorods under visible light. Mater. Lett..

[48] Lu P., Wu J., Shen X., Gao X., Shi Z., Lu M., Yu W.W., Zhang Y. (2020). ZnO–Ti_3_C_2_ MXene Electron Transport Layer for High External Quantum Efficiency Perovskite Nanocrystal Light-Emitting Diodes. Adv. Sci..

[49] Xu Y., Wu R., Zhang J., Shia Y., Zhang B. (2013). Anion-exchange synthesis of nanoporous FeP nanosheets as electrocatalysts for hydrogen evolution reaction. Chem. Commun..

[50] Popczun E.J., McKone J.R., Read C.G., Biacchi A.J., Wiltrout A.M., Lewis N.S., Schaak R.E. (2013). Nanostructured Nickel Phosphide as an Electrocatalyst for the Hydrogen Evolution Reaction. J. Am. Chem. Soc..

[51] Tian L., Yan X., Chen X. (2016). Electrochemical Activity of Iron Phosphide Nanoparticles in Hydrogen Evolution Reaction. ACS Catal..

[52] McEnaney J.M., Crompton J.C., Callejas J.F., Popczun E.J., Read C.G., Lewis N.S., Schaak R.E. (2014). Electrocatalytic hydrogen evolution using amorphous tungsten phosphide nanoparticles. Chem. Commun..

[53] Callejas J.F., Read C.G., Roske C.W., Lewis N.S., Schaak R.E. (2016). Synthesis, Characterization, and Properties of Metal Phosphide Catalysts for the Hydrogen-Evolution Reaction. Chem. Mater..

[54] Li C., Gao H., Wan W., Mueller T. (2019). Mechanisms for hydrogen evolution on transition metal phosphide catalysts and a comparison to Pt (111). Phys. Chem. Chem. Phys..

[55] Shao Y., Shi X., Pan H. (2017). Electronic, Magnetic, and Catalytic Properties of Thermodynamically Stable Two-Dimensional Transition-Metal Phosphides. Chem. Mater..

[56] Cheng Z., Zhang X., Zhang H., Liu H., Yu X., Dai X., Liu G., Chen G. (2020). Ti_2_P monolayer as a high performance 2-D electrode material for ion batteries. Phys. Chem. Chem. Phys..

[57] Liu Q., Xing J., Jiang Z., Jiang X., Wang Y., Zhao J. (2020). 2D tetragonal transition-metal phosphides: An ideal platform to screen metal shrouded crystals for multifunctional applications. Nanoscale.

[58] Kadioglu Y. (2021). Ballistic transport and optical properties of a new half-metallic monolayer: Vanadium phosphide. Mater. Sci. Eng. B.

[59] Blöchl P.E. (1994). Projector augmented-wave method. Phys. Rev. B.

[60] Kresse G., Joubert D. (1999). From ultrasoft pseudopotentials to the projector augmented-wave method. Phys. Rev. B.

[61] Kresse G., Furthmüller J. (1996). Efficient iterative schemes for ab initio total-energy calculations using a plane-wave basis set. Phys. Rev. B.

[62] Kresse G., Hafner J. (1993). Ab initio molecular dynamics for liquid metals. Phys. Rev. B.

[63] Kresse G., Hafner J. (1994). Ab initio molecular-dynamics simulation of the liquid-metal–amorphous-semiconductor transition in germanium. Phys. Rev. B.

[64] Kresse G., Furthmüller J. (1996). Efficiency of ab-initio total energy calculations for metals and semiconductors using a plane-wave basis set. Comput. Mater. Sci..

[65] Perdew J.P., Burke K., Ernzerhof M. (1996). Generalized Gradient Approximation Made Simple. Phys. Rev. Lett..

[66] Grimme S., Antony J., Ehrlich S., Krieg H. (2010). A consistent and accurate ab initio parametrization of density functional dispersion correction (DFT-D) for the 94 elements H-Pu. J. Chem. Phys..

[67] Monkhorst H.J., Pack J.D. (1976). Special points for Brillouin-zone integrations. Phys. Rev. B.

[68] Sowa H., Ahsbahs H. (2006). High-pressure X-ray investigation of zincite ZnO single crystals using diamond anvils with an improved shape. J. Appl. Cryst..

[69] Schoenberg N. (1954). An X-ray investigation of transition metal phosphides. Acta Chem. Scand..

[70] Rossi L.M., Costa N.J.S., Silva F.P., Wojcieszak R. (2014). Magnetic nanomaterials in catalysis: Advanced catalysts for magnetic separation and beyond. Green Chem..

[71] Biz C., Fianchini M., Gracia J. (2020). Catalysis Meets Spintronics; Spin Potentials Associated with Open-Shell Orbital Configurations Enhance the Activity of Pt_3_Co Nanostructures for Oxygen Reduction: A Density Functional Theory Study. ACS Appl. Nano Mater..

[72] Campbell V.E., Tonelli M., Cimatti I., Moussy J.-B., Tortech L., Dappe Y.J., Rivière E., Guillot R., Delprat S., Mattana R. (2016). Engineering the magnetic coupling and anisotropy at the molecule–magnetic surface interface in molecular spintronic devices. Nat. Commun..

[73] Tang W., Sanville E., Henkelman G. (2009). A grid-based Bader analysis algorithm without lattice bias. J. Phys. Comput. Mater..

[74] Sanville E., Kenny S.D., Smith R., Henkelman G. (2007). An improved grid-based algorithm for Bader charge allocation. J. Comp. Chem..

[75] Henkelman G., Arnaldsson A., Jónsson H. (2006). A fast and robust algorithm for Bader decomposition of charge density. Comput. Mater. Sci..

